# Impact of a Hospital-Level Intervention to Reduce Heart Disease Overreporting on Leading Causes of Death

**DOI:** 10.5888/pcd10.120210

**Published:** 2013-05-16

**Authors:** Teeb Al-Samarrai, Ann Madsen, Regina Zimmerman, Gil Maduro, Wenhui Li, Carolyn Greene, Elizabeth Begier

**Affiliations:** Author Affiliations: Ann Madsen, Regina Zimmerman, Gil Maduro, Wenhui Li, Elizabeth Begier, Bureau of Vital Statistics, New York City Department of Health and Mental Hygiene, New York, New York; Carolyn Greene, Division of Epidemiology, New York City Department of Health and Mental Hygiene, New York, New York.

## Abstract

**Introduction:**

The quality of cause-of-death reporting on death certificates affects the usefulness of vital statistics for public health action. Heart disease deaths are overreported in the United States. We evaluated the impact of an intervention to reduce heart disease overreporting on other leading causes of death.

**Methods:**

A multicomponent intervention comprising training and communication with hospital staff was implemented during July through December 2009 at 8 New York City hospitals reporting excessive heart disease deaths. We compared crude, age-adjusted, and race/ethnicity-adjusted proportions of leading, underlying causes of death reported during death certification by intervention and nonintervention hospitals during preintervention (January–June 2009) and postintervention (January–June 2010) periods. We also examined trends in leading causes of death for 2000 through 2010.

**Results:**

At intervention hospitals, heart disease deaths declined by 54% postintervention; other leading causes of death (ie, malignant neoplasms, influenza and pneumonia, cerebrovascular disease, and chronic lower respiratory diseases) increased by 48% to 232%. Leading causes of death at nonintervention hospitals changed by 6% or less. In the preintervention period, differences in leading causes of death between intervention and nonintervention hospitals persisted after controlling for race/ethnicity and age; in the postintervention period, age accounted for most differences observed between intervention and nonintervention hospitals. Postintervention, malignant neoplasms became the leading cause of premature death (ie, deaths among patients aged 35–74 y) at intervention hospitals.

**Conclusion:**

A hospital-level intervention to reduce heart disease overreporting led to substantial changes to other leading causes of death, changing the leading cause of premature death. Heart disease overreporting is likely obscuring the true levels of cause-specific mortality.

The Government are very keen on amassing statistics. They collect them, add them, raise them to the nth power, take the cube root and prepare wonderful diagrams. But what you must never forget is that every one of those figures comes in the first instance from the chowky dar (village watchman) who just puts down what he damn pleases. *Stamp* (1)

## Introduction

Cause-of-death data are critical for determining public health programs and priorities, and they contribute to clinical and epidemiologic research. In the United States, all deaths and their causes must be reported to local and state authorities, and in most cases, the cause of death is reported by physicians. However, documentation is often incomplete, subjective, or inaccurate, partly because physicians lack training in cause-of-death documentation (2–4). Physicians rarely recognize the implications of cause-of-death documentation for national health statistics or that misclassification can influence public health and research priorities (2,4).

Although heart disease is the leading cause of death worldwide, multiple studies have found that heart disease is overreported as a cause of death on death certificates in the United States (5,6) and abroad (7). When certificates from New York City (NYC) in-hospital deaths in 2003 were compared with medical charts, coronary heart disease had been overreported by 91%. Overreporting increased with age: 51% for decedents aged 35 to 74 years, 94% for those aged 75 to 84 years, and 137% for those aged 85 years or older (8). Because the prevalence of heart disease risk factors in NYC is comparable or better than the prevalence nationally, overreporting likely contributes to NYC’s higher-than-expected proportion of deaths from heart disease, 38% compared with 25% nationwide (9–11). Overreporting also obscures trends in health disparities because the level of overreporting varies by hospital (12,13).

To address this problem, NYC’s Department of Health and Mental Hygiene (DOHMH), Bureau of Vital Statistics, conducted an intervention at 8 NYC hospitals to reduce heart disease overreporting through physician and hospital staff training and outreach (13). Given the decline in heart disease deaths reported after the intervention, we sought to determine the impact of this intervention on reporting of other leading causes of death.

## Methods

We used a preintervention–postintervention analysis to evaluate changes in reporting of leading causes of death other than heart disease following an intervention to educate physicians and hospital staff on how to report cause of death during death registration. The intervention was conducted in 8 NYC hospitals during June 2009 through January 2010.

Of the 64 general hospitals in NYC that reported more than 50 total deaths in 2008, the 8 intervention hospitals reported the highest proportions of heart disease deaths, as described in Madsen et al (13). Combined, these 8 hospitals reported approximately 10% of all NYC deaths in 2008. Seven of the 8 intervention hospitals were teaching hospitals. Four were in Brooklyn; 1 was in the Bronx; 2 in Queens; and 1 in Staten Island. Median hospital size was 224 beds (range, 206–705). The comparison group of nonintervention hospitals consisted of the remaining 56 NYC hospitals that reported more than 50 total deaths in 2008. Nonintervention hospitals were distributed across all 5 boroughs. In 2009, all nonintervention hospitals combined reported 25% of heart disease deaths in NYC (13). The intervention and analysis did not pose any risk to living patients, was conducted as a quality improvement activity, and did not require institutional review board approval per NYC Health Code.

### Outcomes and follow-up

We analyzed NYC death certificate data. Primary outcomes were proportions of deaths attributable to leading underlying causes: cerebrovascular disease, malignant neoplasm, influenza and pneumonia, chronic lower respiratory disease, and heart disease. The underlying cause of death is the “disease or injury that initiated the chain of events leading directly to the death, or the circumstances of the accident of violence which produced the fatal injury” and is determined by applying the standardized *International Classification of Diseases, 10th Revision* (ICD-10) algorithm to the words written in the death certificate cause-of-death section (14). The underlying cause ICD-10 codes are assigned automatically to each death by National Center for Health Statistics’ Mortality Medical Data System software or manually by a person trained in classification of diseases if the software cannot automatically assign a code.

We defined categories of deaths as follows: heart disease deaths include ICD-10 codes I00–I09, I11, I13, or I20–I51; malignant neoplasms deaths, codes C00–C97; influenza and pneumonia deaths, codes J09–J18; cerebrovascular diseases deaths, codes I60–I69; and chronic lower respiratory disease deaths, codes J40–J47 (9,15). To understand any trends in actual cause of death outside of the intervention, we also determined these proportions for nonintervention hospitals. To understand the intervention’s impact on citywide statistics, we evaluated intervention hospital, nonintervention hospital, and citywide proportion of deaths attributable to these causes for 2000 through 2010.

### Statistical analysis

We compared the change in the proportion of deaths attributed to each of the leading causes of death at all intervention hospitals with the proportion at all nonintervention NYC hospitals across two 6-month intervals: the preintervention period, January through June 2009; and the postintervention period, January 2010 through June 2010 (13). The relative change in the proportions of deaths attributed to individual leading causes (X) between preintervention and postintervention periods for intervention and nonintervention hospitals was calculated as follows: [(X_post_/Total deaths_post_) – (X_pre_/Total deaths_pre_)] × 100/(X_pre_/Total deaths_pre_).

We stratified the outcome according to sex and age groups. Proportions of heart disease, malignant neoplasm, and influenza and pneumonia deaths were stratified by age (<35, 35–74, 75–84, and ≥85 years) and race/ethnicity (Hispanic, white non-Hispanic, and black non-Hispanic). The categories Asian/Pacific Islander, other, and unknown race/ethnicity were combined into 1 group for this analysis because their numbers were small. We restricted the subgroup analysis to the 3 leading causes of death because the number of deaths for other causes at intervention hospitals was small. To evaluate whether any changes in the distribution of leading causes of death reflected year-to-year fluctuations at intervention hospitals or regression to the mean, we evaluated crude proportions of deaths attributable to these causes from 2000 through 2010. To control for seasonal differences in cause of death, we restricted this analysis to deaths reported during January through June, the period included in the preintervention and postintervention comparison.

Heart disease mortality and other causes of death vary by age and race/ethnicity (16–18). The age and race/ethnicity composition of populations served by intervention and nonintervention hospitals may vary across hospitals or over time. Therefore, to control for potential confounding caused by geographic and temporal differences in age and race/ethnicity distribution of patient populations at intervention and nonintervention hospitals, we standardized age and race/ethnicity to NYC projected population estimates for 2009 by using direct standardization (19,20). We calculated and compared crude, age-adjusted, and race/ethnicity-adjusted proportions for the 3 leading causes of death at intervention and nonintervention hospitals during the periods immediately preintervention and postintervention. DOHMH’s vital event data reflect a complete census of all deaths occurring in NYC; therefore, we did not perform significance testing or calculate confidence intervals to account for sampling variability.

## Results

Total counts of deaths at intervention hospitals during the preintervention and postintervention periods were 2,120 and 2,069, respectively. As reported in Madsen et al, the proportion of deaths reported as attributable to heart disease at intervention hospitals during the preintervention period ranged from 58% to 80%. During the postintervention period, the proportion of deaths reported as attributable to heart disease had declined by 28 to 53 percentage points, to a range of 26% to 44%.

Table 1 lists the top 5 leading causes of death in intervention and nonintervention hospitals during preintervention and postintervention periods. Postintervention, the proportion of deaths due to each leading cause changed at intervention hospitals but not at nonintervention hospitals. Heart disease deaths at intervention hospitals decreased from 69% of reported deaths preintervention to 32% of reported deaths postintervention, a 54% relative decline (Table 1). Concurrent with this decrease, the other 4 leading causes of death increased. Chronic lower respiratory disease had the greatest relative increase (232%), from 2% to 5% of all intervention hospital deaths. The frequency of influenza and pneumonia deaths nearly tripled, from 4% of all intervention hospital deaths to 11%, representing a 193% increase. The proportion of deaths attributed to cerebrovascular disease doubled, from 2% to 4%. Malignant neoplasms, a more frequent cause of death, increased to a lesser degree, from 11% to 16% of intervention hospital deaths, a 48% increase. In comparison, the changes for leading causes of death at nonintervention hospitals were all 6% or less (Table 1).

**Table 1:** Proportion of Deaths Reported for New York City’s Leading Causes of Death, Preintervention and Postintervention^a^, 2009–2010

Cause of Death	Intervention Hospitals			Nonintervention Hospitals	
	Preintervention, n (%) (n = 2,120)	Postintervention, n (%) (n = 2,069)	Relative Change, %	Preintervention, n (%) (n = 22,644)	Relative Change, %
Heart disease	1,454 (69)	654 (32)	–54	8,471 (37)	–5
Malignant neoplasms	232 (11)	335 (16)	48	5,565 (25)	4
Influenza and pneumonia	80 (4)	229 (11)	193	1,054 (5)	–6
Cerebrovascular disease	39 (2)	82 (4)	115	655 (3)	–1
Chronic lower respiratory disease	34 (2)	110 (5)	232	734 (3)	0

a Preintervention period was January through June 2009; postintervention period was January through June 2010.

The changes observed at intervention hospitals in reporting leading causes of death did not reflect the continuation of a trend begun before the intervention or regression to the mean. For 2000 through 2009, the proportion of heart disease deaths at intervention hospitals during January through June (the same portion of the year used in the preintervention–postintervention analysis) was consistently between 60% and 71% (Figure). Reporting of malignant neoplasm, influenza and pneumonia, and chronic lower respiratory disease deaths fluctuated slightly during this period but reached their highest reported levels during January through June 2010 (postintervention period) at intervention hospitals. This pattern was not observed at nonintervention hospitals.

**Figure:**
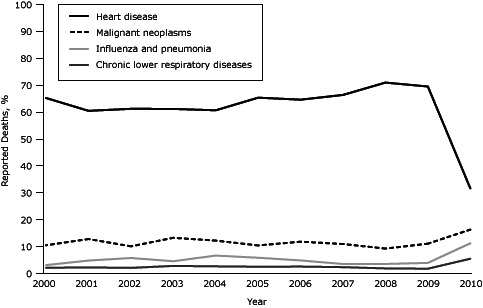
Leading causes of death (as percentage of all deaths) at intervention hospitals, New York City, January–June, 2000–2010. Leading cause of death as listed on New York City death certificates was used for all analyses. All data points are for the January–June period of each given year. **Table:** 

Year	Heart Disease, %	Malignant Neoplasms, %	Influenza and Pneumonia, %	Chronic Lower Respiratory Diseases, %
**2000**	65.3	10.4	2.9	1.95
**2001**	60.5	12.7	4.65	2.1
**2002**	61.3	9.95	5.6	1.9
**2003**	61.2	13.1	4.4	2.6
**2004**	60.7	12.1	6.5	2.5
**2005**	65.4	10.3	5.7	2.3
**2006**	64.7	11.7	4.7	2.4
**2007**	66.4	10.8	3.35	2.2
**2008**	71.1	9.1	3.4	1.7
**2009**	69.6	10.9	3.8	1.6
**2010**	31.6	16.2	11.1	5.3

When analyzed by sex, the rank order of leading causes of death among men and women did not change after the intervention, but the proportions differed (Table 2). Malignant neoplasm deaths were 11% of deaths among both sexes before the intervention but increased to a greater extent among men (to 20%) than women (to 13%) postintervention. Influenza and pneumonia deaths increased to a greater degree among women, from 4% to 12%, than men, from 3% to 10%. We observed no such changes at nonintervention hospitals (Table 3).

**Table 2:** Leading Causes of Death at Intervention Hospitals, Preintervention and PostIntervention^a^, By Sex, Age, and Race/Ethnicity, New York City, 2009–2010^b^

Characteristic	All, n (Column %)		Heart Disease, n (% of Deaths)		Malignant Neoplasm, n (% of Deaths)		Influenza and Pneumonia, n (% of Deaths)	
	Pre	Post	Pre	Post	Pre	Post	Pre	Post
**All**	2,120	2,069	1,454 (69)	654 (32)	232 (11)	335 (16)	80 (4)	229 (11)
**Sex^c^**								
Men	999 (47)	1,025 (50)	671 (67)	331 (32)	112 (11)	195 (20)	32 (3)	97 (10)
Women	1,121 (53)	1,044 (50)	783 (69)	323 (31)	120 (11)	140 (13)	48 (4)	132 (12)
**Age, y**								
≤34	64 (3)	44 (2)	8 (13)	3 (7)	3 (5)	2 (5)	1 (2)	1 (2)
35–74	694 (33)	655 (32)	392 (56)	163 (25)	129 (19)	170 (26)	22 (3)	44 (7)
75–84	543 (26)	536 (26)	376 (69)	162 (30)	71 (13)	97 (18)	25 (5)	66 (12)
≥85	819 (39)	834 (40)	678 (83)	326 (39)	29 (4)	66 (8)	32 (4)	117 (14)
**Age-standardized proportion**			36.0	16.5	11.3	14.9	2.5	4.9
**Race/ethnicity^c^**								
White	1,486 (70)	1,520 (73)	1,064 (72)	499 (33)	148 (10)	244 (16)	61 (4)	187 (12)
Black	257 (12)	252 (12)	165 (64)	76 (30)	27 (11)	39 (16)	6 (2)	22 (9)
Hispanic	214 (10)	95 (4)	127 (59)	27 (28)	26 (12)	9 (10)	5 (2)	7 (7)
Other	163 (8)	202 (10)	98 (60)	52 (26)	31 (19)	43 (21)	8 (5)	13 (6)
**Race-standardized proportion**			64.9	30.0	11.9	14.8	3.3	9.3

a Preintervention period was January through June 2009; postintervention period was January through June 2010.

b Percentages for sex, age, and race/ethnicity categories use total number of deaths in that category (listed in the All columns) as the denominator. Percentages for age and race/ethnicity categories were obtained by direct standardization to the 2009 New York City projected population estimate. Totals may be 1% less than or greater than 100% because of rounding.

c White race/ethnicity denotes non-Hispanic white; black denotes non-Hispanic black; and other includes Asian/Pacific Islander, other, and unknown race/ethnicity.

**Table 3:** Leading Causes of Death at Nonintervention Hospitals, Preintervention and Postintervention^a^, by Sex, Age, and Race/Ethnicity, New York City, 2009–2010^b^

Characteristic	All, n (Column %)		Heart Disease, n (% of Deaths)		Malignant Neoplasm, n (% of Deaths)		Influenza and Pneumonia, n (% of Deaths)	
	Pre	Post	Pre	Post	Pre	Post	Pre	Post
**All**	22,644	22,065	8,471 (37)	7,830 (35)	5,565 (25)	5,618 (25)	1,054 (5)	963 (4)
**Sex^c^**								
Men	11,009 (49)	10,696 (48)	3,885 (35)	3,615 (34)	2,762 (25)	2,724 (25)	462 (4)	459 (4)
Women	11,635 (51)	11,368 (52)	4,586 (39)	4,215 (37)	2,803 (24)	2,894 (25)	592 (5)	504 (4)
**Age, y**								
≤34	928 (4)	919 (4)	48 (5)	40 (4)	91 (10)	71 (8)	23 (2)	17 (2)
35–74	9,553 (42)	9,340 (42)	2,665 (28)	2,421 (26)	3,055 (32)	3,147 (34)	287 (3)	258 (3)
75–84	5,592 (25)	5,085 (23)	2,307 (41)	2,009 (40)	1,459 (26)	1,400 (28)	286 (5)	252 (5)
≥85	6,571 (29)	6,719 (30)	3,451 (53)	3,358 (50)	960 (15)	1,000 (15)	458 (7)	436 (6)
**Age-standardized proportion**			16.4	17.8	20.6	20.5	2.9	2.5
**Race/ethnicity^c^**								
White	10,512 (46)	11,960 (54)	4,558 (43)	4,689 (39)	2,620 (25)	3,116 (26)	522 (5)	561 (5)
Black	6,362 (28)	6,098 (28)	2,164 (34)	1,944 (32)	1,459 (23)	1,488 (24)	236 (4)	197 (3)
Hispanic	4,190 (19)	2,014 (9)	1,228 (29)	613 (30)	1,048 (25)	464 (23)	194 (5)	106 (5)
Other	1,580 (7)	1,993 (9)	521 (33)	584 (29)	438 (28)	550 (28)	102 (6)	99 (5)
**Race-standardized proportion**			35.9	33.7	24.9	25.0	4.8	4.5

a Preintervention period was January through June 2009; postintervention period was January through June 2010.

b Percentages for sex, age, and race/ethnicity categories use total number of deaths in that category (listed under the All columns) as the denominator. Percentages for age and race/ethnicity categories were obtained by direct standardization to the 2009 New York City projected population estimate. Totals may be 1% less than or greater than 100% because of rounding.

c White race/ethnicity denotes non-Hispanic white; black denotes non-Hispanic black; and other includes Asian/Pacific Islander, other, and unknown race/ethnicity.

When restricted to premature deaths (ie, deaths, among those aged 35–74 y), the rank order of leading causes of death at intervention hospitals changed after the intervention (Table 2). Heart disease and malignant neoplasm deaths switched ranking, and malignant neoplasms became the leading cause of premature death at intervention hospitals in the postintervention period (Table 2) similar to the rankings at nonintervention hospitals both preintervention and postintervention (Table 3).

When restricted to deaths of persons aged 75 or older, influenza and pneumonia moved from the third leading cause of death to the second leading cause of death at intervention hospitals (Table 2); this change was not observed at nonintervention hospitals (Table 3).

Controlling for age differences between intervention and nonintervention hospitals by age-standardization, the proportion of heart disease deaths preintervention at intervention hospitals was 36% and 16% among nonintervention hospitals, an absolute difference of 20 percentage points. Postintervention, the absolute age-standardized difference between intervention and nonintervention hospitals was 1 percentage point (17% vs 18%, respectively). Race/ethnicity standardization did not change the cause-of-death proportions at intervention or nonintervention hospitals (Tables 2 and 3).

## Discussion

In 2009, DOHMH implemented a multicomponent intervention to reduce overreporting of heart disease at 8 NYC hospitals. To our knowledge, this is the first study to investigate the impact of reducing heart disease overreporting on other leading causes of death. We found that concomitant with the greater than 50% reduction in heart disease deaths reported at intervention hospitals, there was a proportional increase in other leading causes of death; no prior study has demonstrated this. Given that mortality data are central to public health monitoring and clinical research and that overreporting of heart disease as a cause of death is a documented national problem (5), the substantial changes observed after this intervention reinforce the importance of similar interventions at hospitals with heart disease overreporting to improve clinicians’ cause-of-death certification practices in NYC and possibly in other jurisdictions.

Age and race/ethnicity differences can account for differences in cause-of-death data. Although differences in proportions of leading causes of death between intervention and nonintervention hospitals persisted despite standardization for age and race/ethnicity before the intervention, virtually all of the differences observed between the proportions of expected heart disease, malignant neoplasm, and influenza and pneumonia deaths at intervention and nonintervention hospitals were eliminated in the postintervention period by controlling for age. The consistency between these groups postintervention suggests that reported causes of death at intervention hospitals were more accurate postintervention, because proportions of leading causes of death normalized near existing values at nonintervention hospitals.

The importance of the overall increase in influenza and pneumonia, particularly among persons aged 75 years or older is unclear and should be interpreted with caution. First, rates of reported influenza and pneumonia deaths in NYC are higher than national rates (9,10). A separate DOHMH investigation of an increase in reported influenza and pneumonia deaths during January 2010, at a time when citywide influenza activity was declining, found that 40% of deaths reported as influenza or pneumonia on the death certificate were due to other causes (21). Second, the increase might reflect a greater likelihood of reporting another intermediate cause of death because of uncertainty about the true underlying cause of death. Like heart disease, influenza and pneumonia might represent a common final pathway to death for multiple conditions and does not necessarily represent the underlying cause of death. On this basis, the increase in influenza and pneumonia deaths reported at intervention hospitals during the postintervention period warrants further investigation.

Heart disease overreporting has been documented to increase with age (8); similarly, we found the largest increase in influenza and pneumonia deaths among decedents aged 85 years or older. Several studies have reported that for patients with advanced age, determining the cause of death is particularly difficult (3,8,22). This suggests that inaccurate reporting of cause of death for patients aged 85 or older reflects not only lack of training but also the complexity and challenge of identifying a single underlying condition or clear sequence of events leading to death. Death certificates force physicians to simplify what might be a complex medical situation (23). Therefore, among persons of advanced age who often have multiple comorbidities, the validity and consistency or potential for interrater reliability of cause-of-death data likely declines (24,25). As the population lives longer and multiple chronic medical conditions become more common, documenting which disease is the underlying cause of death will become more challenging. Therefore, using the current 1-cause-to-1-death model for cause-of-death coding might not be appropriate when describing mortality among persons aged 85 or older (24). One consideration is to use contributing and multiple cause-of-death data (ie, all the clinical conditions and events listed on the death certificate) when analyzing mortality data among this age group (26,27).

The poor quality of mortality data is a systemic problem, and the greatest contributor is most likely the limited physician training on death certification. Cause-of-death assignment practices also appear to vary by region (28,29). Other factors that might influence physician practice in NYC are reviewed elsewhere (13). Recommendations from the National Center for Health Statistics of the Centers for Disease Control and Prevention (CDC) on ways to improve national mortality data through physician education on death certification have, for the most part, not been implemented (30). Although the initial NYC intervention was implemented at hospitals with high levels of heart disease reporting, an expansion of the intervention to improve cause-of-death documentation in other hospitals is ongoing in NYC with preliminarily positive results. In addition, completion of an online training module on cause-of-death documentation is now required for NYC physicians using the electronic death registration system (15).

Our study has limitations. First, this was not a validation study; therefore, we did not validate death certificate cause of death before or after the intervention. Second, individual physician understanding of death certification was not formally evaluated postintervention. A validation study is being considered for the future but was too costly and, therefore, not feasible at this time. Of note, Madsen et al’s study determined that the average number of conditions listed in the cause-of death pathway per death certificate increased from 2.4 to 3.4, suggesting that the overall quality and detail on death certificates improved at intervention hospitals (13). Third, standardization for racial/ethnic differences may mask differences in reporting heart disease deaths based on race/ethnicity. Finally, the findings of this study are from 1 urban center and may not be generalizable nationally.

Our findings demonstrate that as overreporting of heart disease deaths declined at intervention hospitals, reporting of other leading causes of death increased substantially at the same hospitals. Because heart disease overreporting also occurs in other hospitals, this study has implications for other jurisdictions, particularly because it suggests underreporting of other diseases (5–7). Mortality data are used extensively to follow disease trends and to determine priorities for epidemiologic, clinical and biomedical research, public health programs, and health care funding allocation, all of which are affected by poor quality cause-of-death data. In NYC, overreporting of heart disease appears to mask the true burden of malignant neoplasms, chronic respiratory diseases, and other causes of death. Consideration should be given to expanding this intervention more broadly, evaluating durability, communicating its impact to stakeholders, and monitoring changes in clinician cause-of-death certification practices. Improvements in cause-of-death reporting may affect our understanding of disease epidemiology and shape public health priorities, policies, and programs.
